# Survival outcomes following interval versus primary debulking surgery in advanced epithelial ovarian cancer: A retrospective cohort study in Lagos, Southwest Nigeria

**DOI:** 10.21203/rs.3.rs-3834135/v1

**Published:** 2024-01-05

**Authors:** Kehinde SHARAFADEEN OKUNADE, Temitope V. Adekanye, Benedetto Osunwusi, Adaiah Soibi-Harry, Austin C. Okoro, Eselobu Toks-Omage, Adebola A. Okunowo, Ephraim O. Ohazurike, Rose I. Anorlu

**Affiliations:** University of Lagos; Lagos University Teaching Hospital; Lagos University Teaching Hospital; Lagos University Teaching Hospital; Lagos University Teaching Hospital; University of Lagos; University of Lagos; Lagos University Teaching Hospital; University of Lagos

**Keywords:** Epithelial ovarian cancer, Lagos, Surgical debulking, Survival, Upfront surgery

## Abstract

**Background:**

There is conflicting evidence regarding the survival benefit of interval debulking surgery (IDS) compared to conventional treatment with primary debulking surgery (PDS) in women with advanced epithelial ovarian cancer (EOC).

**Objectives:**

We compared the survivals following PDS followed by adjuvant chemotherapy (ACT) versus IDS after neoadjuvant chemotherapy (NACT) in women with advanced EOC at the gynaecological oncology unit of a tertiary referral centre in Lagos, Southwest Nigeria.

**Methods:**

The data of 126 women with advanced EOC who had standard treatment with either PDS and ACT or NACT and IDS between January 2008 and December 2017 were analyzed. Kaplan-Meier estimates of progression-free (PFS) and overall survival (OS) time stratified by the types of upfront debulking surgery were calculated and compared by employing the log-rank test statistics. Cox proportional hazard models were then used to estimate hazard ratios of the association between the type of surgical debulking and survivals while adjusting for all necessary covariates.

**Results:**

We recorded no statistically significant differences in PFS (adjusted hazard ratio=1.28, 95% confidence interval 0.82–2.01, P=0.282) and OS (adjusted hazard ratio=1.23, 95% confidence interval 0.68–2.25, P=0.491) between IDS and PDS among women with advanced EOC.

**Conclusions:**

There is a need for a larger prospective multicenter study to further compare the impact of upfront surgical debulking types on the survival of women with advanced EOC in our setting. In the meantime, giving interval debulking surgery after a few courses of neoadjuvant chemotherapy should be an acceptable standard of care for women with advanced EOC.

## Introduction

In 2020, ovarian cancer ranked as the eighth most commonly diagnosed cancer among women, with 313,959 new cases and 207,252 recorded deaths worldwide.^1^ It is the leading cause of death in women diagnosed with gynaecological cancers.^1^ Most cases of ovarian cancer are epithelial in origin^2^ with more than 70–80 % f these cases diagnosed at the later stages thus leading to poor outcomes for patients.^3,4^ This is because early-stage diseases are mostly asymptomatic or characterized by non-specific symptomatology making diagnosis very challenging. Ovarian cancer, therefore, remains an unresolved global health problem.^5,6^

In women with advanced EOC, the extent of residual disease is a critical prognostic factor influencing survival outcomes.^7–9^ Primary debulking surgery (PDS) followed by adjuvant platinum-based chemotherapy (ACT) constitutes the primary treatment approach for ovarian cancer as this treatment aims to eliminate any gross or microscopic residual tumour.^9^ Optimal surgical debulking is not always possible for advanced-stage diseases, especially in women with very extensive diseases beyond the pelvis^9^ or those with a major medical condition that may impair their performance status and inability to withstand an extensive PDS.^10^ In such instances, surgery can be intricate, occasionally necessitating substantial bowel resection and significant blood loss, posing a considerable risk of morbidity and mortality.^9,11,12^ When PDS is not feasible, secondary surgery referred to as interval debulking surgery (IDS) may be carried out after an initial tumour response in between a few courses of chemotherapy. Although there is no optimal timing of IDS, it is usually performed after two to four courses of neoadjuvant chemotherapy (NACT).^9^

IDS after NACT has demonstrated improved tumour resectability and response rates.^13,14^ However, there is still conflicting evidence regarding its survival benefits compared to conventional treatment with PDS followed by adjuvant chemotherapy,^11,12,15^ especially in resource-limited settings including Nigeria. Therefore, this current study compared the survival outcomes (progression-free and overall survivals) in women with advanced EOC who had either PDS and ACT or NACT and IDS over 10 years at the women’s cancer unit of a tertiary referral centre in Nigeria.

## Patients and Methods

### Study design and settings

This was a retrospective cohort study of all advanced epithelial ovarian cancer (EOC) cases that had the standard treatment with either primary debulking surgery (PDS) and adjuvant chemotherapy (ACT) or neoadjuvant chemotherapy (NACT) and interval debulking surgery (IDS) at a gynaecological oncology unit of a referral hospital in Lagos, Southwest Nigeria between January 2008 and December 2017. The hospital is a leading tertiary healthcare facility in Southwest Nigeria, primarily functioning as a specialized referral centre for government-owned and private hospitals in Lagos, Ogun, and Oyo States. The hospital’s gynaecologic oncology unit comprising four consultant gynaecologic oncologists and up to 15 resident doctors provides various in-patient and out-patient multidisciplinary oncology services including the screening and treatment of different premalignant and malignant diseases of the female genital tract.^16^

### Eligibility criteria

We retrieved data from the medical records of women diagnosed and treated for EOC in the women’s cancer unit from January 2008 to December 2017. We also extracted data on tumour recurrence and death for each patient for up to three years of follow-up after completion of first-line treatment until December 2020. The inclusion criteria were: patients with the surgico-pathological diagnosis of advanced EOC defined as the International Federation of Gynecology and Obstetrics (FIGO) stages 3 and 4;^17^ women who received complete standard treatment consisting of either PDS and postoperative ACT or preoperative NACT, IDS and postoperative ACT; and those with complete medical records and laboratory test results required in the final data analyses. Women who did not complete their treatment were excluded from the study.

### Study procedure and data collection

We included n = 126 women with the surgico-pathological diagnosis of advanced EOC data in the study. We retrieved data from the gynaecological oncology ward register and patients’ medical records such as age, menstrual status, parity, body mass index (BMI), coexisting morbidity (such as hypertension, diabetes mellitus, kidney, and liver disease), presence of ascites, preoperative serum CA-125 levels and tumour recurrence and timing of recurrence. Obesity was defined as a BMI greater than or equal to 30 kg/m^2^.^18^ We defined tumour recurrence as the clinical and/or radiologic evidence of tumour regrowth after a period of remission or death within three years of completion of standard first-line treatment.

### Study outcomes

We evaluated two study endpoints: progression-free survival (PFS), characterized as the duration from the conclusion of primary treatment to the initial indication of disease progression, as determined by clinical examination, elevated tumour marker (serum CA125 levels), and/or radiological studies; and overall survival (OS), defined as the duration from the completion of primary treatment to death from any cause or the last follow-up for patients still alive after completing treatment. Survival data were censored after a three-year follow-up period.^19^

### Statistical analyses

Data analyses were performed using SPSS version 28.0 for Windows (IBM Corp., Armonk, NY, USA) and descriptive statistics were computed for patients’ relevant sociodemographic, and clinical characteristics. Baseline patient characteristics were described using mean and standard deviation (for normally distributed variables) or median and interquartile range (for skewed variables) for continuous parameters. Categorical variables were presented using frequencies and percentages. Kaplan-Meier estimates of PFS and OS time, stratified by the types of upfront debulking surgery, were computed and compared using log-rank test statistics.^20^ Cox proportional hazard models using backward stepwise conditional techniques were then used to estimate hazard ratios (HR) of the association between the type of surgical debulking and survivals (PFS and OS) while adjusting for the participants’ clinicopathologic characteristics. Variables with *P* < 0.20 were built into the final multivariable models. Statistical significance for associations was reported when the *P*-value was less than 0.05.

### Ethical considerations

The research protocol for this study was approved by the Research Ethics Committee of the hospital (ADM/DCST/HREC/APP/3699) according to the Declaration of Helsinki before access to the patient’s medical records and subsequent data collection. Strict confidentiality measures were maintained for patients’ information throughout and following the conclusion of the study.

## Results

**Figure 1:**
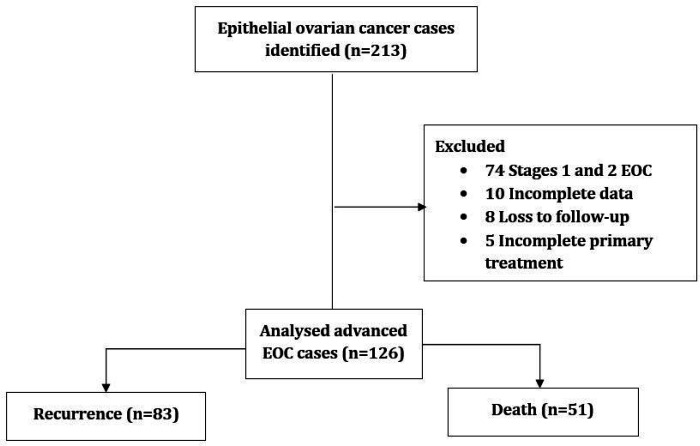
Study patients’ flow chart

We retrieved the data of n = 213 women diagnosed with epithelial ovarian cancer (EOC) and managed with the standard first-line treatment during the review period. Based on the study eligibility, the datasets of n = 126 women who had advanced EOC were included in the final data analyses [Figure 1].

The mean age of women in the study was 51.1 ± 13.3 years, mean BMI was 25.1 ± 5.2 kg/m^2^, and median serum CA-125 levels of 467 (246, 1550) U/mL. The women were predominantly multiparous (n = 79, 62.7%), premenopausal (n = 66, 52.4%), and had no pre-existing medical condition (n = 96, 76.2%). Other patients’ baseline characteristics are presented in Table 1. There are statistically significant differences in the patients’ mean age (P = 0.019), mean serum CA 125 levels (P < 0.001), presence of comorbidity (P = 0.019), FIGO disease stage (P = 0.002) and debulking surgery status (P = 0.001) between women who had IDS and those who had PDS as their upfront surgical treatment.

**Figure 2:**
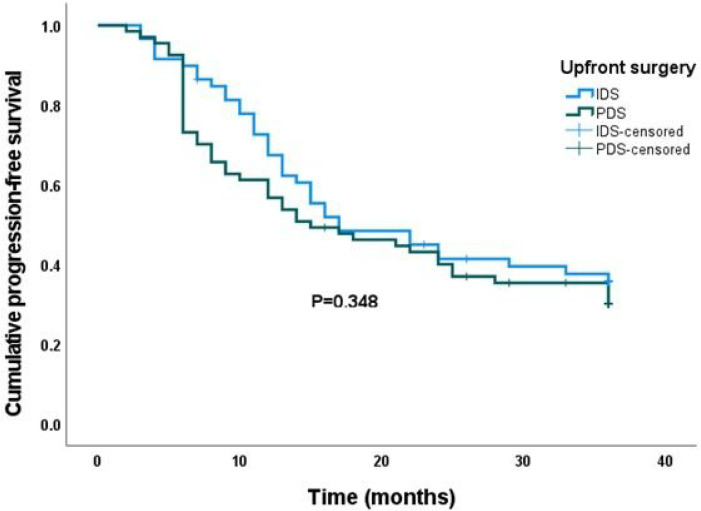
Kaplan-Meier curve of progression-free survival (PFS) stratified by type of upfront debulking surgery. There was no significant difference in PFS between interval and primary debulking surgery (P =0.348).

As shown in Fig. 2, the Kaplan–Meier survival analysis recorded an overall median progression-free survival (PFS) of 17.0 months (95% confidence interval 10.8, 23.2). There was no statistically significant difference in median PFS after three years between women who had interval debulking surgery after preoperative neoadjuvant chemotherapy and those who had primary debulking surgery followed by postoperative adjuvant chemotherapy (17.0 vs. 15.0 months, P = 0.348). As shown in Table 2, the majority of the women in the study had PDS as their upfront surgical treatment (n = 67, 53.2%). Tumour recurrence was recorded in 83 (65.9%) women during the period under review. Following adjustments for age, parity, BMI, co-morbidity and tumour histological subtype, there was no statistically significant difference in PFS between women in the IDS arm and those in the PDS arm (adjusted hazard ratio = 1.28, 95% confidence interval = 0.82–2.01, P = 0.282).

**Figure 3:**
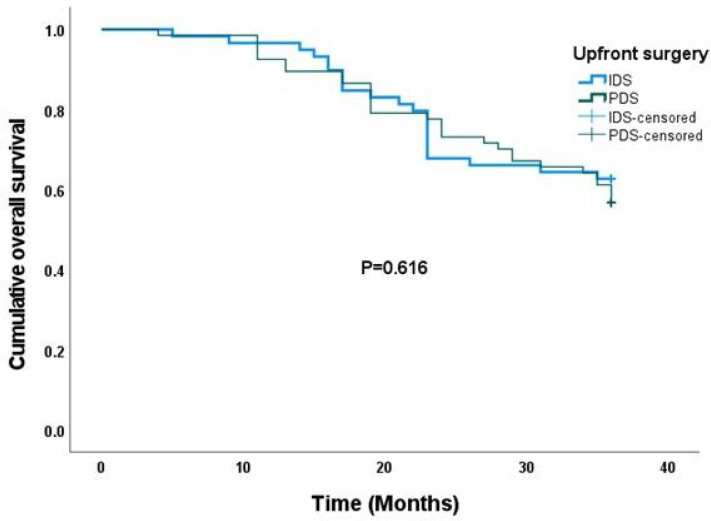
Kaplan-Meier curve of overall survival (OS) stratified by type of upfront debulking surgery. There was no significant difference in OS between interval and primary debulking surgery (P =0.348).

In Fig. 3, the Kaplan–Meier survival analysis showed a median overall survival (OS) of 30.1 months (95% confidence interval 28.6, 31.7). There was no statistically significant difference in median OS after three years between women who had interval debulking surgery and those who had primary debulking surgery as their first-line upfront surgical treatment (30.0 vs. 30.2 months, P = 0.616). Fifty-one of the 126 women (40.5%) in the study died within three years of completion of their primary treatment. On adjustments for covariates including menstrual status, BMI, co-morbidity, FIGO stage and presence of ascites, there was no statistically significant difference in the OS between women in the IDS arm and those in the PDS arm (adjusted hazard ratio = 1.23, 95% confidence interval 0.68–2.25, P = 0.491) [Table 3].

## Discussion

Investigations have indicated that an advanced FIGO stage independently worsened PFS^21–23^ and OS^24^ in patients with EOC. On this premise, our current study examined the impact of the type of upfront debulking surgery on these survival outcomes by comparing the survivals of women with advanced EOC who had interval debulking surgery after neoadjuvant chemotherapy with women who had the standard first-line treatment with primary debulking surgery followed by adjuvant chemotherapy. We recorded no significant differences in survival outcomes among women in both treatment groups.

The impact of early tumour recurrence on the overall survival of patients with EOC is widely acknowledged.^25^ In this study, we recorded a tumour recurrence rate of 65.9%, which is much lower than the rate of 100% recorded in a similar cohort of women with advanced EOC in a previous study conducted in the same setting in Lagos, Nigeria.^23^ The rate is however lower than the disease recurrence rate of over 70% within the first 5 years reported in most series involving women with advanced EOC who achieved a full remission following first-line therapy.^8,26^ In addition, the relatively high death rate recorded in this study may point to the category of women included in the study which further suggests that an advanced stage may be associated with poor survival outcomes.^23^ However, the variation in study findings, especially with regards to recurrence, may be due to the different follow-up periods adopted in our study and that of previous studies as the risk of disease recurrence and death increases with increasing duration since completion of first-line treatment.

Studies have reported that the primary independent prognostic factor in advanced ovarian cancer is the complete removal of all macroscopic disease during debulking surgery.^27,28^ A difficult optimal resection of macroscopic disease due to pelvic and peritoneal fibrosis after neoadjuvant chemotherapy has been hypothesized as a potential drawback of upfront IDS.^15^ However, this was not corroborated by our current study where women who had IDS compared to PDS have an unadjusted higher optimal surgical debulking rate (44.1% versus 17.9%). This is similar to previous studies that showed that women with advanced FIGO stage of EOC who had NACT followed by IDS showed statistically and clinically improved optimal cytoreduction,^12,15^ perioperative complications, and mortality^29,30^ compared to patients who had PDS and postoperative ACT. However, these did not translate to differences in survival benefits as our current study and previous randomized controlled trials conducted by Vergote et al. in 2010^15^ and Kehoe et al. in 2015^12^ reported that survival with IDS is similar to PDS in women with stage III or IV EOC. An almost similar finding to our study was also reported in a retrospective study conducted in Slovenia and published by Kobal et al. in 2018^31^ that showed no significant differences in terms of PFS (17.3 versus months) and OS (41.3 versus 34.5 months) in the PDS and IDS arms. This thus suggests that no differences in survival duration in women with advanced ovarian diseases regardless of the geographical settings where treatment is received.

These findings draw attention to the findings from the Gynecologic Oncology Group (GOG)^32^ and European Organization for Research and Treatment of Cancer (EORTC)^33^ trials that suggested the need for optimal tumour response to chemotherapy before IDS. This is because women with advanced diseases in these trials whose tumour masses were chemo-reduced to less than 1 cm before IDS had better survival than women who had PDS followed by chemotherapy. Even though the associations between upfront surgery type and survival outcomes in our study were not influenced by the disease debulking status, we suggest that the potential of a woman with either FIGO stage 3 or 4 diseases to respond to an initial chemo-reduction should still be used as a criterion for selecting treatment that would achieve optimal survival benefits.

There are some limitations in this study. First, there are potential selection biases limited by the single-centre study. Second, the retrospective design of the study with no power calculation could result in inadequate sample size thus limiting the statistical power of the study. Third, the restriction of the study follow-up period to three years instead of the standard survival assessment after five years could make comparison to previous studies difficult. Fourth, we did not assess the influence of baseline performance status on the type of treatment selection and the introduction of second-line treatment during follow-up and their consequent impact on patients’ survival data.^24^ Finally, there could be variations in the expertise and/or the level of training of surgeons who performed the upfront debulking surgical procedures during the review period which could have some influence on our reported findings. However, our study is the first to examine survival outcomes based on upfront surgical treatment in women with advanced EOC in sub-Saharan Africa, and therefore, our finding would add to the growing body of literature in this important area of ovarian cancer management.

## Conclusions

We found no evidence that IDS between cycles of chemotherapy compared with conventional treatment using PDS followed by postoperative ACT improved survivals in women with advanced EOC. We, therefore, suggest the need for a larger prospective multicenter study to further compare the impact of upfront surgical debulking types on the survival of women with advanced EOC in our setting with adjustments for important covariates that may influence survival such as patients’ baseline performance status and use of second-line treatment during follow-up. In addition, this study should examine the adverse effects, quality of life, or postoperative morbidity or mortality profiles of these treatment modalities. In the meantime, giving interval debulking surgery after a few courses of neoadjuvant chemotherapy should be an acceptable standard of care for women with advanced EOC.

## Figures and Tables

**Table 1:** Patients baseline characteristics (n = 126)

Characteristics	Type of upfront surgical treatment		P-value
	IDS (n = 59)	PDS (n = 67)	
Mean age (± SD) in years	48.5 ± 13.8	53.4 ± 12.5	0.019
Mean BMI (± SD) in kg/m^2^	25.2 ± 5.7	25.1 ± 4.7	0.429
Median CA-125 levels (IQR) in U/mL	370 (144, 1000)	606 (370, 1935)	< 0.001
Parity			
Nulliparity	21 (35.6%)	26 (38.8%)	0.710
Multiparity	38 (64.4%)	41 (61.2%)	
Menstrual status			
Premenopause	35 (59.3%)	31 (46.3%)	0.143
Postmenopause	24 (40.7%)	36 (53.7%)	
Pre-existing morbidity			
Yes	19 (32.2%)	11 (16.4%)	0.038
No	40 (67.8%)	56 (83.6%)	
Ascites			
Yes	34 (57.6%)	37 (55.2%)	0.786
No	25 (42.4%)	30 (44.8%)	
FIGO stage			
Stage 3	48 (81.4%)	37 (55.2%)	0.002
Stage 4	11 (18.6%)	30 (44.8%)	
Debulking surgery status			
Optimal	26 (44.1 %)	12 (17.9%)	0.001
Suboptimal	33 (55.9%)	55 (82.1%)	
Histological subtype			
Type I (LGSC and others)	28 (47.5%)	18 (26.9%)	0.017
Type II (HGSC)	31 (52.5%)	49 (73.1%)	

Abbreviations: CA, cancer antigen; FIGO, International Federation of Gynecology and Obstetrics; HGSC, high-grade serous carcinomas; IDS, interval debulking surgery; IQR, interquartile range; LGSC, low-grade serous carcinomas; PDS, primary debulking surgery; SD, standard deviation.

**Table 2:** Univariable and multivariable analyses of type of upfront surgery and progression-free survival

Factors	Number of women with recurrence within 3 years	Crude	Adjusted	
		P-value	HR (95% CI)	P-value
**Type of upfront surgery**				
IDS	37/59 (62.7%)	0.396	1.28 (0.82–2.01)	0.282
PDS	46/67 (68.7%)		1.00 (reference)	
Age				
< 52 years	34/62 (54.8%)	0.005	1.87 (1.19–2.93)	0.006
≥ 52 years	49/64 (76.6%)		1.00 (reference)	
Parity				
Nulliparity	25/47 (53.2%)	0.015	1.42 (0.85–2.37)	0.180
Multiparity	58/79 (73.4%)		1.00 (reference)	
Menopausal status				
Premenopause	41/66 (62.1%)	0.523	NA	NA
Postmenopause	42/60 (70.0%)			
BMI				
Obese (≥ 30 kg/m^2^)	19/23 (82.6%)	0.044	0.62 (0.36–1.06)	0.078
Non-obese (< 30 kg/m^2^)	64/103 (62.1%)		1.00 (reference)	
Serum CA-125 levels				
≥ 470 U/mL	39/61 (63.9%)	0.588	NA	NA
< 470 U/Ml	44/65 (67.7%)			
Pre-existing morbidity				
Yes	23/30 (76.7%)	0.262	NA	NA
No	60/96 (62.5%)			
Presence of ascites				
Yes	48/71 (67.6%)	0.263	NA	NA
No	35/55 (63.6%)			
FIGO stage				
Stage 3	54/85 (63.5%)	0.313	NA	NA
Stage 4	29/41 (70.7%)			
Surgical debulking status				
Optimal	28/38 (73.7%)	0.175	0.63 (0.39–1.01)	0.053
Suboptimal	55/88 (62.5%)		1.00 (reference)	
Histological subtype				
Type I	27/46 (58.7%)	0.305	NA	NA
Type II	56/80 (70.0%)			

Abbreviations: BMI, body mass index; CA, cancer antigen; CI, confidence interval; FIGO, International Federation of Gynecology and Obstetrics; HR, hazard ratio; NA, not applicable; PDS; primary debulking surgery; Type I includes low-grade serous carcinomas and others; Type II includes high-grade serous carcinomas.

**Table 3:** Univariable and multivariable analyses of type of upfront surgery and overall survival

Factors	Number of women that died within 3 years	Crude	Adjusted	
		P-value	HR (95% CI)	P-value
**Type of upfront surgery**				
IDS	22/59 (37.3%)	0.622	1.23 (0.68–2.25)	0.491
PDS	29/67 (43.3%)		1.00 (reference)	
Age				
< 52 years	26/62 (41.9%)	0.884	NA	NA
≥ 52 years	25/64 (39.1%)			
Parity				
Nulliparity	17/47 (36.2%)	0.272	NA	NA
Multiparity	34/79 (43.0%)			
Menopausal status				
Premenopause	33/66 (50.0%)	0.032	0.65 (0.36–1.18)	0.156
Postmenopause	18/60 (30.0%)		1.00 (reference)	
BMI				
Obese (# x2265; 30 kg/m^2^)	4/23 (17.4%)	0.036	3.58 (1.28–10.02)	0.015
Non-obese (< 30 kg/m^2^)	47/103 (45.6%)		1.00 (reference)	
Serum CA-125 levels				
≥ 470 U/mL	21/61 (34.4%)	0.254	NA	NA
< 470 U/mL	30/65 (46.2%)			
Pre-existing morbidity				
Yes	10/30 (33.3%)	0.672	NA	NA
No	41/96 (42.7%)			
Presence of ascites				
Yes	34/71 (47.9%)	0.055	0.49 (0.27–0.87)	0.016
No	17/55 (30.9%)		1.00 (reference)	
FIGO stage				
Stage 3	30/85 (35.3%)	0.117	1.40 (0.78–2.52)	0.259
Stage 4	21/41 (51.2%)		1.00 (reference)	
Surgical debulking status				
Optimal	15/38 (39.5%)	0.943	NA	NA
Suboptimal	36/88 (40.9%)			
Histological subtype				
Type I	20/46 (43.5%)	0.798	NA	NA
Type II	31/80 (38.8%)			

Abbreviations: BMI, body mass index; CA, cancer antigen; CI, confidence interval; FIGO, International Federation of Gynecology and Obstetrics; HR, hazard ratio; NA, not applicable; PDS; primary debulking surgery; Type I includes low-grade serous carcinomas and others; Type II includes high-grade serous carcinomas.

## References

[1] Sung H, Ferlay J, Siegel RL, Global Cancer Statistics 2020: GLOBOCAN Estimates of Incidence and Mortality Worldwide for 36 Cancers in 185 Countries. CA Cancer J Clin. 2021;71(3):209–249. doi:10.3322/caac.21660 33538338

[2] Kaku T, Ogawa S, Kawano Y, Histological classification of ovarian cancer. Medical Electron Microscopy. 2003;36(1):9–17. doi:10.1007/s007950300002 12658347

[3] Minig L, Zorrero C, Iserte PP, Poveda A. Selecting the best strategy of treatment in newly diagnosed advanced-stage ovarian cancer patients. World J Methodol. 2015;5(4):196–202. doi:10.5662/wjm.v5.i4.196 26713279 PMC4686416

[4] Okunade KS, Adetuyi IE, Adenekan M, Ohazurike E, Anorlu RI. Risk predictors of early recurrence in women with epithelial ovarian cancer in lagos, nigeria. Pan African Medical Journal. 2020;36. doi:10.11604/pamj.2020.36.272.17827 PMC7546017 33088401

[5] Torre LA, Trabert B, DeSantis CE, Ovarian cancer statistics, 2018. CA Cancer J Clin. 2018;68(4):284–296. doi:10.3322/caac.21456 29809280 PMC6621554

[6] Nash Z, Menon U. Ovarian cancer screening: Current status and future directions. Best Pract Res Clin Obstet Gynaecol. 2020;65:32–45. doi:10.1016/j.bpobgyn.2020.02.010 32273169

[7] Rizzuto I, Stavraka C, Chatterjee J, Risk of Ovarian Cancer Relapse score: a prognostic algorithm to predict relapse following treatment for advanced ovarian cancer. Int J Gynecol Cancer. 2015;25(3):416–422. doi:10.1097/IGC.0000000000000361 25647256 PMC4340599

[8] du Bois A, Reuss A, Pujade-Lauraine E, Harter P, Ray-Coquard I, Pfisterer J. Role of surgical outcome as prognostic factor in advanced epithelial ovarian cancer: a combined exploratory analysis of 3 prospectively randomized phase 3 multicenter trials: by the Arbeitsgemeinschaft Gynaekologische Onkologie Studiengruppe Ovarialkarzinom (AGO-OVAR) and the Groupe d’Investigateurs Nationaux Pour les Etudes des Cancers de l’Ovaire (GINECO). Cancer. 2009;115(6):1234–1244. doi:10.1002/cncr.24149 19189349

[9] Tangjitgamol S, Manusirivithaya S, Laopaiboon M, Lumbiganon P, Bryant A. Interval debulking surgery for advanced epithelial ovarian cancer. Cochrane Database Syst Rev. 2013;(4):CD006014. doi:10.1002/14651858.CD006014.pub6 23633332 PMC4161115

[10] Okunade KS, Soibi-Harry AP, Osunwusi B, Preoperative Predictors of Optimal Tumor Resectability in Patients With Epithelial Ovarian Cancer. Cureus. Published online January 19, 2022. doi:10.7759/cureus.21409 PMC8855641 35198316

[11] Fagotti A, Ferrandina G, Vizzielli G, Phase III randomised clinical trial comparing primary surgery versus neoadjuvant chemotherapy in advanced epithelial ovarian cancer with high tumour load (SCORPION trial): Final analysis of peri-operative outcome. Eur J Cancer. 2016;59:22–33. doi:10.1016/j.ejca.2016.01.017 26998845

[12] Kehoe S, Hook J, Nankivell M, Primary chemotherapy versus primary surgery for newly diagnosed advanced ovarian cancer (CHORUS): an open-label, randomised, controlled, non-inferiority trial. Lancet. 2015;386(9990):249–257. doi:10.1016/S0140-6736(14)62223-6 26002111

[13] Hou JY, Kelly MG, Yu H, Neoadjuvant chemotherapy lessens surgical morbidity in advanced ovarian cancer and leads to improved survival in stage IV disease. Gynecol Oncol. 2007;105(1):211–217. doi:10.1016/j.ygyno.2006.11.025 17239941

[14] Fagö-Olsen CL, Ottesen B, Kehlet H, Does neoadjuvant chemotherapy impair long-term survival for ovarian cancer patients? A nationwide Danish study. Gynecol Oncol. 2014;132(2):292–298. doi:10.1016/j.ygyno.2013.11.035 24321400

[15] Vergote I, Tropé CG, Amant F, Neoadjuvant chemotherapy or primary surgery in stage IIIC or IV ovarian cancer. N Engl J Med. 2010;363(10):943–953. doi:10.1056/NEJMoa0908806 20818904

[16] Okunade KS, John-Olabode S, Ohazurike EO, Soibi-Harry A, Osunwusi B, Anorlu RI. Predictors of early mortality risk in patients with epithelial ovarian cancer. Health Sci Rep. 2022;5(4). doi:10.1002/hsr2.717 PMC9260216 35821892

[17] Berek JS, Renz M, Kehoe S, Kumar L, Friedlander M. Cancer of the ovary, fallopian tube, and peritoneum: 2021 update. International Journal of Gynecology & Obstetrics. 2021;155(S1):61–85. doi:10.1002/ijgo.13878 34669199 PMC9298325

[18] Weir CB, Jan A. BMI Classification Percentile And Cut Off Points.; 2023. 31082114

[19] Okunade KS, John-Olabode SO, Soibi-Harry AP, Prognostic performance of pretreatment systemic immune-inflammation index in women with epithelial ovarian cancer. Future Sci OA. 2023;9(10):FSO897. doi:10.2144/fsoa-2023-0108 37753357 PMC10518822

[20] Kaplan EL, Meier P. Nonparametric Estimation from Incomplete Observations. Vol 53.; 1958.

[21] Liu XH, Man YN, Wu XZ. Recurrence Season Impacts the Survival of Epithelial Ovarian Cancer Patients. Asian Pacific Journal of Cancer Prevention. 2014;15(4):1627–1632. doi:10.7314/APJCP.2014.15.4.1627 24641379

[22] Yan XJ, Liang LZ, Zeng ZY, Liu JH, Yuan SH, Wei M. [Recurrence risk factors of platinum-sensitive epithelial ovarian cancer]. Ai Zheng. 2005;24(6):751–754. 15946495

[23] Sharafadeen Okunade K, Adejimi AA, Ohazurike EO, Predictors of Survival Outcomes After Primary Treatment of Epithelial Ovarian Cancer in Lagos, Nigeria. Published online 2021. doi:10.1200/GO.20 PMC8081541 33449803

[24] Andreou M, Kyprianidou M, Cortas C, Prognostic Factors Influencing Survival in Ovarian Cancer Patients: A 10-Year Retrospective Study. Cancers (Basel). 2023;15(24):5710. doi:10.3390/cancers15245710 38136256 PMC10742060

[25] Chan JK, Tian C, Teoh D, Survival after recurrence in early-stage high-risk epithelial ovarian cancer: a Gynecologic Oncology Group study. Gynecol Oncol. 2010;116(3):307–311. doi:10.1016/j.ygyno.2009.10.074 19944452

[26] Schwab CL, English DP, Roque DM, Pasternak M, Santin AD. Past, present and future targets for immunotherapy in ovarian cancer. Immunotherapy. 2014;6(12):1279–1293. doi:10.2217/imt.14.90 25524384 PMC4312614

[27] Giorda G, Gadducci A, Lucia E, Prognostic role of bowel involvement in optimally cytoreduced advanced ovarian cancer: a retrospective study. J Ovarian Res. 2014;7:72. doi:10.1186/1757-2215-7-72 25328074 PMC4100746

[28] Trifanescu OG, Gales LN, Trifanescu RA, Anghel RM. Clinical prognostic factors in pre- and post-menopausal women with ovarian carcinoma. Acta Endocrinol (Buchar). 2018;14(3):353–359. doi:10.4183/aeb.2018.353 31149283 PMC6525767

[29] Hou JY, Kelly MG, Yu H, Neoadjuvant chemotherapy lessens surgical morbidity in advanced ovarian cancer and leads to improved survival in stage IV disease. Gynecol Oncol. 2007;105(1):211–217. doi:10.1016/j.ygyno.2006.11.025 17239941

[30] Morrison J, Haldar K, Kehoe S, Lawrie TA. Chemotherapy versus surgery for initial treatment in advanced ovarian epithelial cancer. Cochrane Database Syst Rev. 2012;(8):CD005343. doi:10.1002/14651858.CD005343.pub3 PMC4050358 22895947

[31] Kobal B, Noventa M, Cvjeticanin B, Primary debulking surgery versus primary neoadjuvant chemotherapy for high grade advanced stage ovarian cancer: comparison of survivals. Radiol Oncol. 2018;52(3):307–319. doi:10.2478/raon-2018-0030 30210049 PMC6137361

[32] Rose PG, Nerenstone S, Brady MF, Secondary surgical cytoreduction for advanced ovarian carcinoma. N Engl J Med. 2004;351(24):2489–2497. doi:10.1056/NEJMoa041125 15590951

[33] van der Burg ME, van Lent M, Buyse M, The effect of debulking surgery after induction chemotherapy on the prognosis in advanced epithelial ovarian cancer. Gynecological Cancer Cooperative Group of the European Organization for Research and Treatment of Cancer. N Engl J Med. 1995;332(10):629–634. doi:10.1056/NEJM199503093321002 7845426

